# 

**Published:** 1992-03

**Authors:** 


### WEST OF ENGLAND MEDICAL JOURNAL

**MARCH 1992** **Volume 107(i)**

Copyright © West of England Medical Journal Ltd 1991.

**CONTENTS**

5 **What's New from The British Journal of Surgery.**
J. R. Farndon

8 **Ethics in Transplantation.**
R. M. R. Taylor

10 **Survey of Services to Patients in General Practice.**
Nicholas Bradley, Rupert Gude

13 **An Audit of the Use of Barium Meal Examinations in General Practice.**
J. E. Baldwin

15 **Obituary — Henry Dendy Moore (1912-1992).**

16 **Correspondence.**

17 **From our Correspondents.**

17 **Crossword.**

19 **What is this Life if Full of Care.**
Peter M. Dunn

24 **Book Reviews.**
Medical Secrets — Zollo
Gastroenterology — Harding, Robinson
Radiology of Trauma — Sclafani
Radiology of the Extemities — Baker

25 **South West Paediatric Club.**

26 **The Place for Paediatricians.**
Thomas E. Oppe

28 **Wessex Branch of The Association of Clinical Pathologists.**

31 **Solutions to Crossword.**

32 **South West Radiologists Association.**

